# The dynamics of the nursing workforce: insights into retention and exit using registration data.

**DOI:** 10.23889/ijpds.v7i3.1896

**Published:** 2022-08-25

**Authors:** Michelle Jamieson

**Affiliations:** 1 Edinburgh Napier University

## Objectives

The Nursing and Midwifery Council (NMC) require data to be collected to enable regulatory functions, with nurses re-registering every 3-years, which provides a powerful picture of the workforce. This could answer questions to inform the NMC, as well as policy makers. Linked to other data, opportunities for impact are considerable.

## Approach

This pilot project, funded by the Economic and Social Research Council, aims to use NMC data captured at two time points – March 2018 to give a snapshot before the pandemic, and March 2021 during the pandemic - in research that enables a better understanding of the dynamics of the nursing profession in the UK, pre- and hopefully moving towards post-pandemic. An observational study using anonymised administrative data collected and held by the NMC as part of the registration and revalidation process on approximately 700,000 registrants across the United Kingdom.

## Results

This emerging pilot study will report the initial results as to likelihood of ceasing registration by relation age, gender, and geographically.

## Conclusion

This pilot study will give an indication of the groups within the nursing profession who are at particular risk of leaving. Findings will provide an opportunity to understand the potential of NMC registrant data to enable future studies into the workforce as well as workforce policy implications.

